# Polyphyly and gene flow between non-sibling *Heliconius *species

**DOI:** 10.1186/1741-7007-4-11

**Published:** 2006-04-21

**Authors:** Vanessa Bull, Margarita Beltrán, Chris D Jiggins, W Owen McMillan, Eldredge Bermingham, James Mallet

**Affiliations:** 1 Galton Laboratory, Department of Biology, University College London, 4 Stephenson Way, London NW1 2HE, UK; 2 Smithsonian Tropical Research Institute, Apartado 2072, Balboa, Panama; 3 Departamento de Biología, Universidad de Puerto Rico, P.O.Box 23360, San Juan, PR 00931-3360, Puerto Rico; 4 Institute of Cell, Animal & Population Biology, University of Edinburgh, King's Buildings, West Mains Road, Edinburgh EH9 3JT, UK

## Abstract

**Background:**

The view that gene flow between related animal species is rare and evolutionarily unimportant largely antedates sensitive molecular techniques. Here we use DNA sequencing to investigate a pair of morphologically and ecologically divergent, non-sibling butterfly species, *Heliconius cydno *and *H. melpomene *(Lepidoptera: Nymphalidae), whose distributions overlap in Central and Northwestern South America.

**Results:**

In these taxa, we sequenced 30–45 haplotypes per locus of a mitochondrial region containing the genes for *cytochrome oxidase *subunits I and II (*CoI*/*CoII*), and intron-spanning fragments of three unlinked nuclear loci: *triose-phosphate isomerase *(*Tpi*), *mannose-6-phosphate isomerase *(*Mpi*) and *cubitus interruptus *(*Ci*) genes. A fifth gene, *dopa decarboxylase *(*Ddc*) produced sequence data likely to be from different duplicate loci in some of the taxa, and so was excluded. Mitochondrial and *Tpi *genealogies are consistent with reciprocal monophyly, whereas sympatric populations of the species in Panama share identical or similar *Mpi *and *Ci *haplotypes, giving rise to genealogical polyphyly at the species level despite evidence for rapid sequence divergence at these genes between geographic races of *H. melpomene*.

**Conclusion:**

Recent transfer of *Mpi *haplotypes between species is strongly supported, but there is no evidence for introgression at the other three loci. Our results demonstrate that the boundaries between animal species can remain selectively porous to gene flow long after speciation, and that introgression, even between non-sibling species, can be an important factor in animal evolution. Interspecific gene flow is demonstrated here for the first time in   *Heliconius* and may provide a route for the transfer of switch-gene adaptations for Müllerian mimicry. The results also forcefully demonstrate how reliance on a single locus may give an erroneous picture of the overall genealogical history of speciation and gene flow.

## Background

Introgression can play a key evolutionary role in plants [1,2], but has until recently been considered rare and controversial in animals [3-5]. This is largely because hybrids between animal species are rare, but may also stem from a belief that hybridization and introgression between species is unnatural [1,3,5]. Hybrids are usually rare on a per individual basis, but species undergoing occasional interpecific hybridization are common. Existing surveys suggest that around 10% of animal species, and 25% of plant species hybridize [5]: for instance, 9% of bird species [6] and 11% of European butterfly species [7] are known to hybridize with at least one other species. Even if occasional, natural hybridization can lead to successful introgression, with important consequences in ecology, evolution and conservation [3,5]. However, hybridization in nature does not guarantee that genes will pass between species, because hybrids are typically selected against, and may be completely infertile or inviable. To determine whether hybridization leads to introgression, we must investigate the patterns of distribution of alleles among hybridizing species. Shared alleles in descendent species may have been inherited as pre-existing polymorphisms from their joint ancestors, as well as via recent gene flow since speciation. These two routes to allelic sharing, which both result in genealogical polyphyly at the species level, are hard to distinguish on the basis of genetic data.

Recently, two classes of molecular methods have been used to test for introgression. Both rely on the idea that introgression in some genomic locations will be prohibited by reproductive isolation or divergent natural selection, while at other loci introgressing alleles will establish more freely. In fact, without heterogeneity of divergence across the genome, it will typically be difficult to discriminate recent speciation from recent gene flow. The first method examines genotypic data at multiple low-resolution loci, such as chromosomal morphs, allozymes, microsatellites, amplified fragment length polymorphisms (AFLPs) or single nucleotide polymorphisms (SNPs), for heterogeneity of divergence in allele frequency. Alleles that flow freely will have their frequencies homogenised across species, while alleles whose introgression is blocked by divergent selection will retain strong frequency differences. Thus, heterogeneity in allele frequency differences among loci suggests that on-going gene flow as a likely explanation for similar allele frequencies at some genes in pairs of taxa that hybridise [8-13]. A second approach, adopted in this paper, employs DNA sequence data, coupled with a statistical approach based on gene genealogies and coalescence theory, to test whether shared haplotype polymorphisms could have been inherited from a common ancestor or are more likely due to introgression since speciation [14-18].

*Heliconius *butterflies are a rapidly radiating, tropical group, well known for diverse warning colors and Müllerian mimicry [19-22]. Around 35% of the species in this group are known to hybridize in nature [5,21]. This study concentrates on *Heliconius cydno *and *H. melpomene*, which co-occur throughout Central America and the Northern Andes [23,24]. Although closely related, separating approximately 1.5 million years ago [24], they are not classical 'sibling species' (i.e. species difficult to distinguish using morphology). Nor are they host races, ecological morphs or members of a recent island radiation. Unusually for studies of introgression, we have chosen two species that are partially sympatric, continental taxa differing strongly in morphology, as well as in larval and adult ecology [21,25,26]. These species diverged in colour pattern due to Müllerian mimicry [27]: *Heliconius melpomene *mimics the geographically widespread red, yellow and black *H. erato*, while *H. cydno *mimics the black and white or yellow *H. sapho *and *H. eleuchia *[27,28] in the Northern Andes and Central America. The two are not even strictly sister species, since a number of other morphologically distinct species, such as *H. heurippa, H. pachinus*, *H. timareta*, and *H. tristero*, appear more closely related to *H. cydno *than to *H. melpomene *[23,29-32]. Nonetheless, natural hybrids occur in most areas of range overlap. The frequency of hybridization is rare (less than one in every 1000 individuals [21,27]), but hybrids of both sexes are viable, and, although F1 hybrid females are sterile, in accordance with Haldane's Rule, males are fertile and produce viable backcross progeny with either parental species [33-35]. Furthermore, about half the hybrids collected from the wild are backcrosses, identifiable by their colour pattern phenotype [35]. Introgression mediated by fertile hybrids therefore seems likely [5,21].

To test the importance of introgressive gene flow between the two species, we sequenced multiple haplotypes of four unlinked loci using a geographic sampling regime designed to reveal unusual genealogical patterns due to introgression. We chose sympatric populations of *H. cydno chioneus *and *H. melpomene rosina *from Panama, which are known from a few specimens to hybridize occasionally in the wild, and an allopatric French Guiana population of *H. melpomene melpomene *that does not overlap with *H. cydno*, and whose mtDNA is also known to be divergent relative to other *H. melpomene *populations [23]. The French Guiana population acts as a control for population divergence and the high variance of coalescence time expected. If introgression were occurring at a particular locus, we would expect sympatric haplotypes to be unusually similar, while at the same time the allopatric population of *H. melpomene *can be used to confirm that evolutionary divergence occurs normally at the same locus. We sequenced DNA from four unlinked loci: one mitochondrial region (*cytochrome oxidase *subunits *I *and *II *– *CoI*, *CoII*; hereafter *Co*), and intron-spanning regions of a sex-linked locus *Tpi *(*triose-phosphate isomerase*, on the *Z*-chromosome), autosomal *Mpi *(*mannose-6-phosphate isomerase*), and *Ci *(*cubitus interruptus*); for details see Table 1). These loci were selected because it was reasoned that mitochondrial genes and nuclear non-coding regions would diverge rapidly enough to provide high resolution between closely related taxa [36]. The nuclear loci are all known to be on different linkage groups [37], providing a scatter of loci across the genome with which to detect introgression.

## Results

### The genealogical pattern at each locus

**Figure 1:**
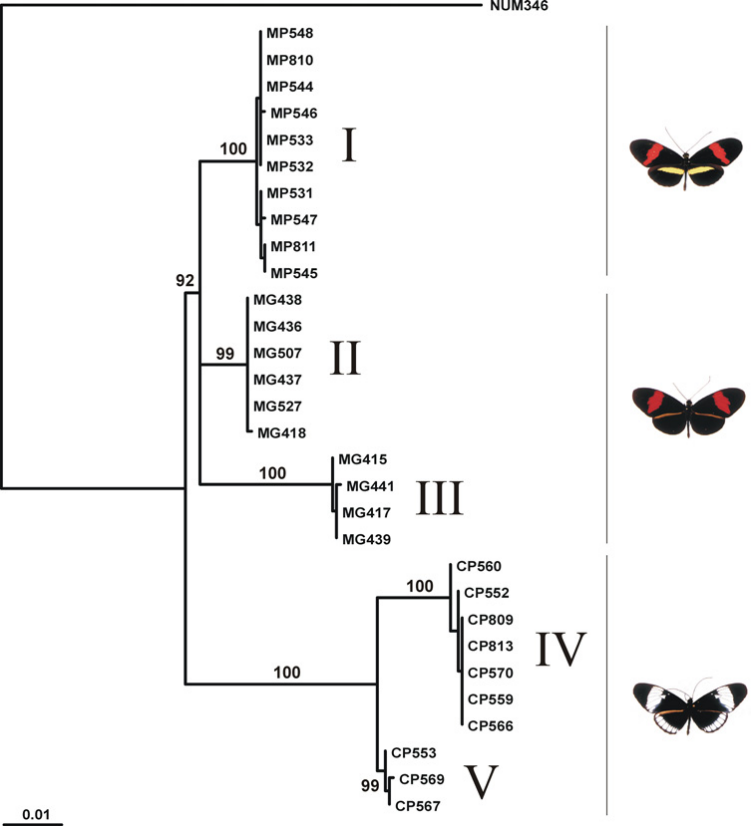
**Maximum likelihood genealogy for *CoI* and *CoII***. Parsimony bootstrap values (>70%) are given above the nodes, taken from the equivalent nodes on the parsimony trees, when available. Major groups of haplotypes, mostly supported by high bootstrap values or indels are identified using Roman numerals. MG = *H. melpomene melpomene *(French Guiana), MP = *H. melpomene rosina *(Panama), CP = *H. cydno chioneus *(Panama), NUM = *H. numata*.

Details of the data and models of evolution are given in Table 1. As expected if allelic coalescence has occurred more recently than the split between the two species, the maximum likelihood genealogy for *Co *is consistent with reciprocal monophyly for *H. melpomene *and *H. cydno*, with 2.5–3.0% net divergence between species compared to < 1.3% uncorrected divergence within populations, and 0.4% net divergence between *H. melpomene *races (Table 2). There was no evidence for recombination at this locus (Table 1), as expected for mitochondrial sequences [38]. A previous parsimony study of *Co *found French Guiana (MG) and Panama (MP) haplotypes of *H. melpomene *to be distinct (as here), and suggested that the French Guiana clade was sister to a clade including Panama *H. melpomene *and *H. cydno *[23], making *H. melpomene *paraphyletic with respect to *H. cydno *(CP) at this locus. Our results differ in that although *melpomene *paraphyly has a marginally greater likelihood, mutual monophyly cannot be rejected (SH test: ΔlogL = 1.36, P = 0.27). Paraphyly of *H. melpomene*, as found by Brower [23] using a parsimony analysis, is however strongly rejected by equal-weighted parsimony bootstrapping on our data. The difference between the two analyses is probably due to additional information provided by the greater length of mitochondrial sequence we used (1573 bp vs. Brower's 942 bp). We therefore show the constrained, mutually monophyletic tree (Fig. 1).

**Figure 2:**
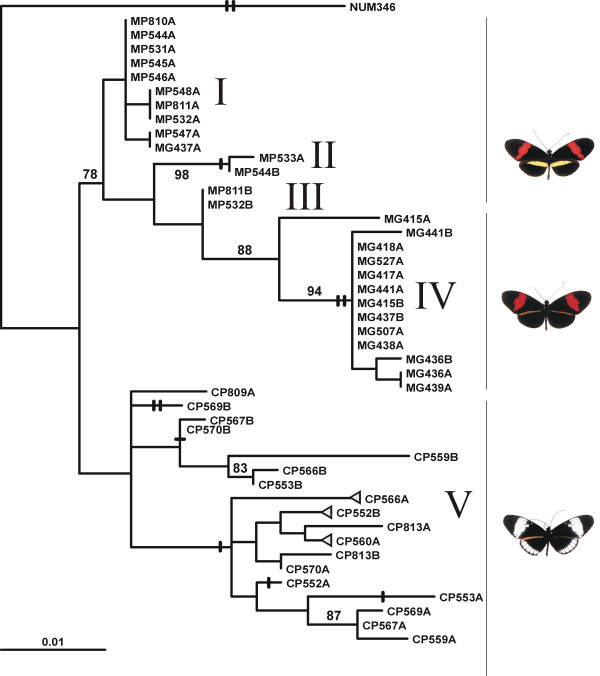
**Maximum likelihood genealogy for *Tpi***. Parsimony bootstrap values (> 70%) are given above the nodes, taken from the equivalent nodes on the parsimony trees, when available. Insertions or deletions (indels) inferred to be apomorphies are shown using black bars. Indels inferred to be homoplasious or to involve reversals not concordant with the given topologies, are shown as triangles. Major groups of haplotypes, mostly supported by high bootstrap values or indels are identified using Roman numerals. MG = *H. melpomene melpomene *(French Guiana), MP = *H. melpomene rosina *(Panama), CP = *H. cydno chioneus *(Panama), NUM = *H. numata*.

The genealogical pattern for *Tpi *is somewhat similar in some respects to that for *Co*, in that mutual monophyly of *H. cydno *and *H. melpomene *seems likely, with 1.4–2.5% net divergence between the two species at this locus, compared to 1.3% between races within *H. melpomene *(Table 2). There are several examples of homoplasious indels in the tree, as expected if recombination had taken place within these haplotypes. There is additional evidence from a statistical test for recombination in both *H. melpomene *(*P *= 0.023), and *H. cydno *(*P *= 0.004) from Panama. In contrast to *Co*, *Tpi *yields an almost reverse maximum likelihood genealogy in which *H. melpomene *forms a monophyletic group within a paraphyletic *Heliconius cydno*. But again, the evidence for paraphyly does not stand up: there is less than 50% parsimony bootstrap support for a paraphyletic tree over a monophyly-constrained tree, and likelihood support for paraphyly is also weak (SH test, ΔlogL = 6.63, P = 0.22). Therefore, we again represent the genealogy as the most likely mutually monophyletic species tree (Fig. 2).

**Figure 3:**
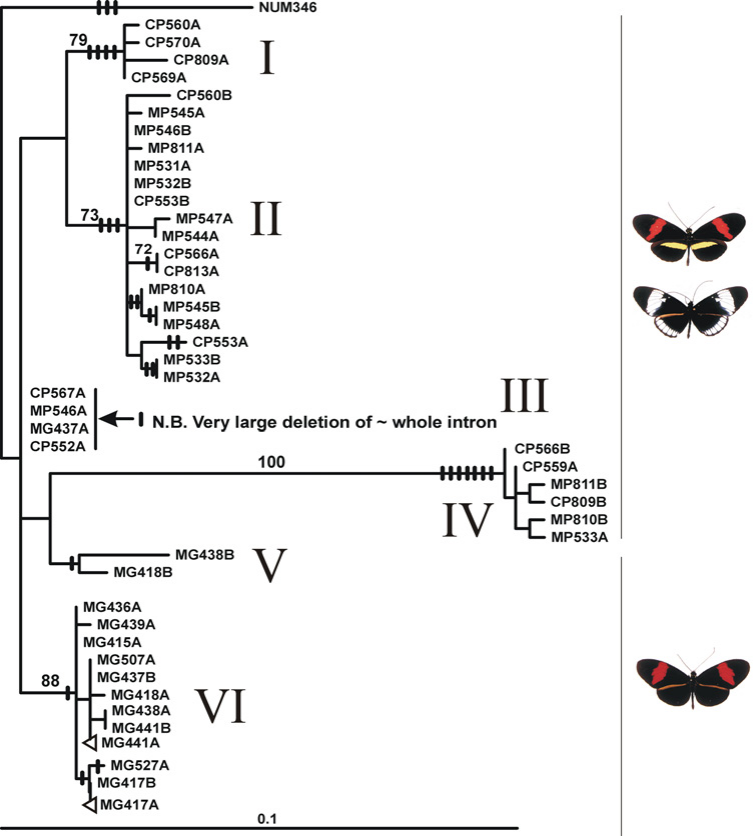
**Maximum likelihood genealogy for *Mpi***. Parsimony bootstrap values (>70%) are given above the nodes, taken from the equivalent nodes on the parsimony trees, when available. Insertions or deletions (indels) inferred to be apomorphies are shown using black bars. Indels inferred to be homoplasious or to involve reversals not concordant with the given topologies, are shown as triangles. Major groups of haplotypes, mostly supported by high bootstrap values or indels are identified using Roman numeral. MG = *H. melpomene melpomene *(French Guiana), MP = *H. melpomene rosina *(Panama), CP = *H. cydno chioneus *(Panama), NUM = *H. numata*.

In contrast, *Mpi *demonstrates a clear lack of reciprocal monophyly between *H. cydno *and *H. melpomene*: net divergence between these species within Panama (0.03%) is much less than between French Guiana and Panama populations of *H. melpomene *(1.91%, Table 2). Two indels inferred to be homoplasious are found in French Guiana *H. melpomene*, suggesting that recombination occurs and a test for recombination confirms this (P = 0.004). However, no evidence for recombination was found in *H. melpomene*, *H. cydno*, or combined haplotypes from Panama (P > 0.25, Table 1). The vast majority of indels conform to the estimated genealogy, suggesting recombination is rare (Fig. 3). The maximum likelihood genealogy suggests that many identical or nearly identical sequences are shared between the two species in Panama, both of which populations are strongly differentiated from *H. melpomene *from French Guiana (Fig. 3). Four very short *Mpi *sequences, with a 280 bp deletion spanning most of the intron, were also identical across taxa (Fig. 3, haplotype group III). These sequences are placed basally by maximum likelihood because of the lack of phylogenetic information. A tree search constrained to be mutually monophyletic between the two species is strongly rejected (ΔlogL = 133.24, P < 0.001).

The existence of some very short alleles and high rates of divergence among haplotype groups within each taxon might suggest that divergent duplicate loci are being sequenced. However, a battery of tests confirm that *Mpi *acts as a single-copy nuclear, coding marker which can be mapped readily in crosses (see methods). Similar results have recently been obtained with the same primers by a number of other workers, showing that the results here are not anomalous [24,39].

The genealogical pattern for *Ci *is more complex. As with *Mpi*, net divergence is low between *H. cydno *and *H. melpomene *in Panama (0.7%), and rather higher between geographic races of *H. melpomene *(3.7%) and between *H. cydno *and French Guiana H. melpomene (2.9%). However, there are no fixed differences between any pair of taxa, and the numbers of shared polymorphisms appear to be roughly equal between all comparisons, both within and between species at this locus (Table 3).

**Figure 4:**
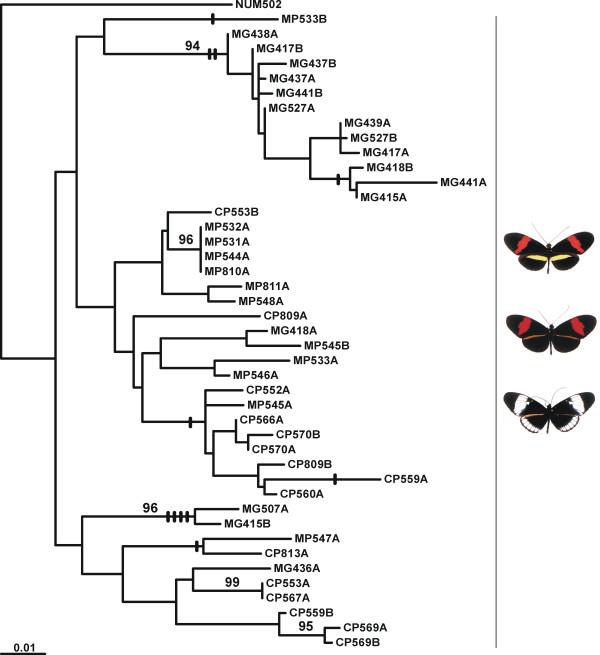
**Maximum likelihood genealogy for *Ci***. Parsimony bootstrap values (>70%) are given above the nodes, taken from the equivalent nodes on the parsimony trees, when available. Insertions or deletions (indels) inferred to be apomorphies are shown using black bars, but indels inferred to be homoplasious or to involve reversals not concordant with the given topologies, are not shown since approximately 27 homoplasious indels would have required showing over 90 gains and losses on the genealogy). This estimated genealogy is poorly resolved and shows many homoplasies, almost certainly due to abundant recombination, and therefore sequence groups were not labeled. MG = *H. melpomene melpomene *(French Guiana), MP = *H. melpomene rosina *(Panama), CP = *H. cydno chioneus *(Panama), NUM = *H. numata*.

Unlike *Mpi*, *Ci *shows many inferred homoplasies, both of single base pairs and of indels: in addition to the apomorphic indels shown, 90 inferred insertion/deletion events involve 27 homoplasious indels, too numerous to be shown in Fig. 4. These homoplasious events are presumably mainly due to recombination (significant in all four separate tests, P = 0.02, P = 0.001, P < 0.001, P < 0.001; see Table 1), which negates the validity of representing the *Ci *genealogy as a strictly bifurcating tree as in the likelihood analysis (Fig. 4). Indels are expected to be less prone to reversal or homoplasy than single-base changes. At the *Ci *locus, seven out of 11 indels not subject to homoplasy or reversal supported groups of French Guiana *melpomene melpomene*, while of the remaining three, two are autapomorphic, one supports a (*cydno*+*melpomene rosina*) haplotype pair. One indel supports a grouping of *H. cydno *with a single associated *H. melpomene *sequence that lacks the indel (but which is attached to the base of the clade, and so does not contravene the idea that the indel may be a character found only in *H. cydno*). These contrasting patterns suggest either multiple ancestral polymorphisms, or introgression of haplotypes and recombination both within and between the two species in Panama, but a lack of gene flow between either of these and *H. melpomene melpomene *sequences from French Guiana. Several groups of *Ci *haplotypes from French Guiana *H. melpomene melpomene *form distinct clades, but overall there is strong evidence against mutually monophyletic separation between *cydno *and *melpomene *(Fig. 4, ΔlogL = 130.63, P < 0.001). Thus the pattern seems similar to that for *Mpi*, but closely similar or identical haplotypes at this locus are rarer between than within species. Thus, if introgression explains similarities among haplotypes of the sympatric species pair, it may be more ancient than at *Mpi*.

### Bayesian analysis of genealogy and introgression

**Figure 5:**
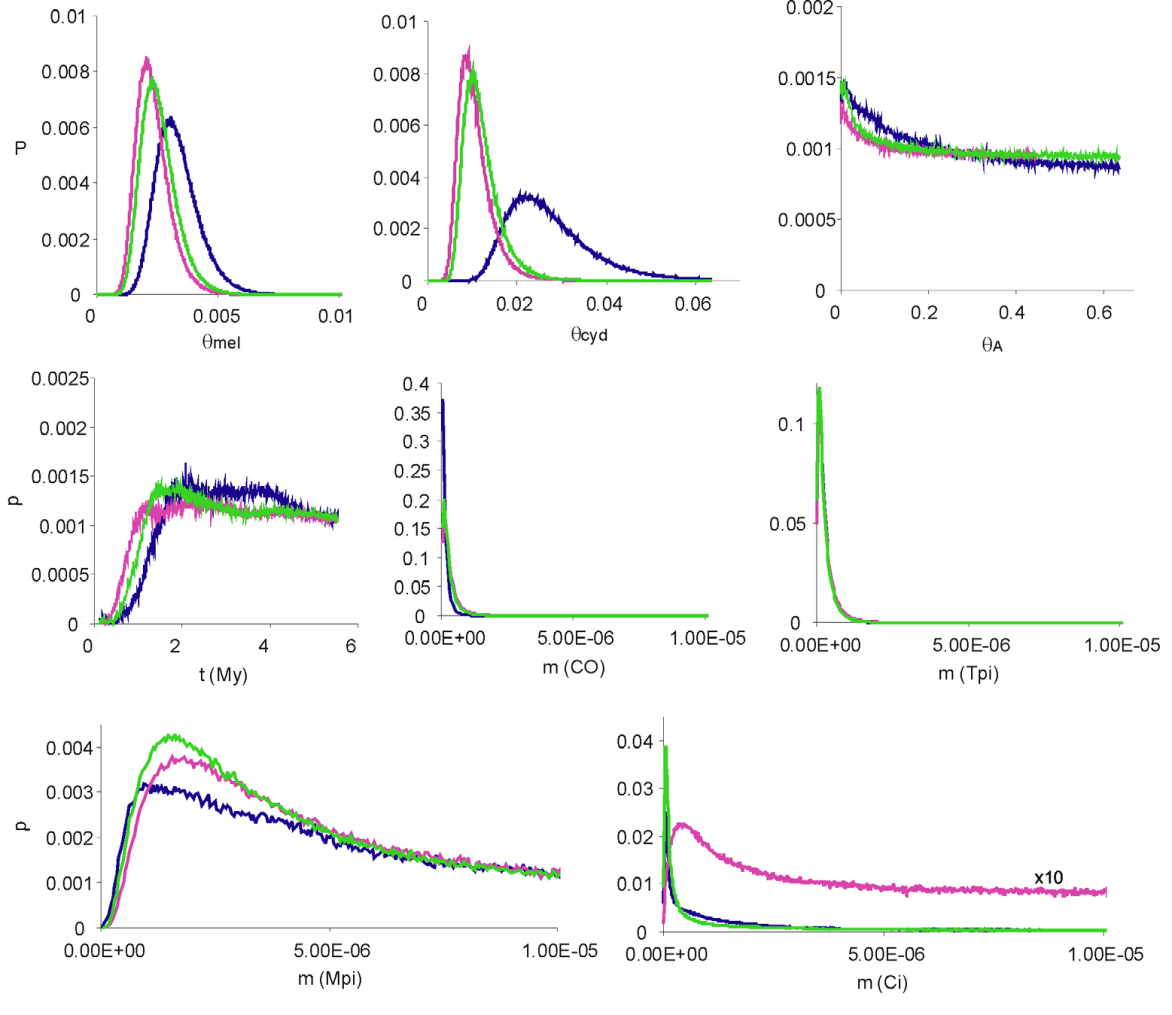
**IM analysis of Panama *H. melpomene *and *H. cydno *data**. The panels show approximate Bayesian posterior probability distributions for effective population size of *H. melpomene *(*θ*_*mel*_), *H. cydno *(*θ*_*cyd*_), and the ancestor of the two species (*θ*_*A*_). The time since divergence of the two species (*t*), and the locus-specific bidirectional introgression rates are also shown (*m*). The three datasets analysed are the basic IM dataset (blue), and IM reduced dataset 1 (pink) and IM reduced dataset 2 (green). Analysis of the basic IM dataset is compromised by recombination within *Tpi *and *Ci*, which is assumed not to occur in the IM algorithm. Reduced datasets containing apparently non-recombined segments of the genes were analyzed to overcome this difficulty. IM reduced dataset 1 differs only from IM reduced dataset 2 in that a different, shorter, part of the *Ci *locus is used; the low sequence information probably explains why there is little information in the former run in the last panel (probabilities for IM dataset 1 are enhanced 10-fold in this panel only, for clarity). The curves show useful parameter estimation *, except in the case of ancestral population size *(*θ*_*A*_), the upper tail of the time of divergence (*t*) and the introgression for *Ci *for IM reduced dataset 1.

The strong polyphyly of *Mpi *and *Ci *genealogies between the two species suggests that selective introgression at these loci may be the cause. To examine this possibility we employed the Bayesian 'Isolation-Migration' (IM) algorithm of Hey & Nielsen [18]. The IM test deals with pairs of taxa only, so we examined only sympatric *H. melpomene rosina *and *H. cydno chioneus *from Panama. We carried out analyses on three modified datasets to conform with the assumptions and limitations of the IM algorithm (i.e. no gap polymorphism, no recombination within loci). Firstly, we obtained a dataset that is as complete as possible obtained by deleting highly indel-laden haplotypes and indel polymorphic regions (the basic 'IM Dataset') – for example, the short intronless sequences at *Mpi *could not be used in this analysis. Two additional datasets were sampled from this basic dataset, by removing apparent recombinant regions or haplotypes ('IM Reduced Dataset 1', and 'IM Reduced Dataset 2' – see methods and Table 1). In the event, all three analyses gave broadly similar results. We calibrate the analyses via neutral substitution rates obtained from an insect mitochondrial DNA clock calibration [32] to obtain approximate per base pair neutral substitution rates (μ) for each locus, times since speciation (*t*), effective population size measured as a product of mutation rate and total population size (θ = 4 *N*μ), and per locus bidirectional introgression rates (*m*). The absolute values of these parameters are of course of some interest, but are highly dependent on the clock calibration of Brower [32] based on a shorter sequence of mtDNA, which may therefore be somewhat unreliable. What are of more interest here are the relative values for introgression (*m*) between the species at different loci. The results are shown in Tables 4, 5, 6 and Fig. 5.

Overall, the results suggest that substitution rates (μ) for the three nuclear loci are three to four-fold higher than the rate for the mitochondrial region. This may seem rapid for nuclear genes, but the comparison is between mitochondrial coding sequence and largely intronic nuclear sequence (Tables 4, 5, 6), and the result agrees approximately with a broader comparative study in *Heliconius *in which third base pairs in codons of the *Co *mitochondrial region were shown to diverge at approximately the same rates as intronic sequences in two of the nuclear genes studied here (*Tpi *and *Mpi*), overall, and apparently even faster at low divergences similar to those found here (1–2% at *Co*; see Figs. 1a, b in ref. [24]). Only weak information is available from the genealogical data about time of divergence, but speciation is inferred to have taken place more than about a million years ago (Tables 4, 5, 6, Fig. 5) and most likely around 2.0 million years ago. Effective population sizes (θ) are estimated to be about three to four-fold larger in *H. cydno *than *H. melpomene rosina*, but the latter species' population size would undoubtedly have been estimated to be larger if the more divergent *H. melpomene melpomene *sequences had been included. Using the IM reduced datasets as better estimates of population size without recombination within each locus, the overall inferred effective population sizes correspond to about 230,000–340,000 individuals for *H. melpomene rosina*, and 1.41–1.75 million individuals for *H. cydno *using the mitochondrial rate calibration of Brower [32]. Very little information is available to estimate the ancestral population size (θ_*A*_), as can be seen from the graphs in Fig. 5.

Of most importance here, bidirectional introgression rates (*m*) for each locus apparently identify a single outlier, *Mpi*, which has *m *at least an order of magnitude higher than at any of the other loci. The lower tails of the posterior probability distributions of *m *for *Co*, *Tpi*, and *Ci *are never complete even at the lowest values sampled, so we follow Hey & Nielsen [18] in inferring a lack of evidence for introgression at such loci.

## Discussion

### The genealogies obtained compared with other available data

The maximum likelihood genealogy for the *Co *region, based on all *H. cydno *and *H. melpomene *and outgroup data now available from GenBank (62 sequences, data not shown) suggests paraphyly of *H. melpomene*, but with an Amazonian/French Guiana clade of *H. melpomene *outside a paired sister group consisting of a clade of Western South American, Central American and French Guiana *H. melpomene*, and a clade containing *H. cydno*. Nonetheless, monophyly is again not rejected overall (ΔlogL = 1.74, P = 0.19), and parsimony bootstrap support for paraphyly of *melpomene *is < 50%. Similarly, monophyly is not rejected at *Tpi*, even when all available GenBank data for this locus (101 sequences, data not shown) are taken into consideration. For *Mpi*, in contrast, genealogical polyphyly remains highly supported when we employ data from the entire GenBank record (92 sequences, data not shown). For instance, a recent study from a different laboratory demonstrated sequences from *melpomene *in each of the haplotype groups I-II and IV of Fig. 3[39]. The presence of *H. melpomene *sequences within haplotype group I is especially significant as only *H. cydno *haplotypes are found in this group in the present study (Fig. 3), almost certainly due to the small number of haplotypes we sampled in group I. No other data from these taxa are yet available for the *Ci *locus. In conclusion, all other available data lend further support to the genealogical patterns we find here.

### Gene flow or ancestral polymorphism?

Why have *Mpi *and *Ci *not acquired fixed differences since speciation of *H. melpomene *and *H. cydno*, while *CoI*/*CoII *and *Tpi *have done so? Two major explanations seem possible: (1) retention of ancestral *Mpi *and *Ci *polymorphism, and lack of its retention in *CoI*/*CoII *and *Tpi*, or (2) selective introgression of *Mpi *and/or *Ci *alleles. Ancestral polymorphism initially seems plausible because the effective population size (*N*_*e*_) should be higher for autosomal *Mpi *and *Ci *than for sex-linked *Tpi *and maternally inherited *CoI*/*CoII*. However, given a 1:1 sex ratio, ratios of *N*_*e *_among the three gene regions (4:3:1 respectively) are not large: for example, average coalescence time for *Mpi *or *Ci *should be only 1.33-fold of that of *Tpi *on this basis. Zero introgression explanations would also require extremely low rates of substitution within each ancestral *Mpi *allelic group (I-IV in Fig. 3) to explain the presence of identical and near-identical intron sequences (e.g. Panama melpomene 546B, 531A, 532B vs. cydno 553B; Panama *melpomene *811B vs. cydno 809B; Panama melpomene 546A vs. cydno 567A and 552A) in species that split over 1 million years ago. However, lack of divergence seems highly unlikely, because rates of divergence at *Mpi *and *Ci *are approximately three times faster than rates at *CoI*/*CoII *(Tables 4, 5, 6) as estimated here, and when tested among more distantly related species of *Heliconius *[24]. Also divergence is normal between geographic populations of *H. melpomene *– see below. Thus, even if the *Mpi *haplotype groups I-IV had been retained as ancestral polymorphisms, we would have expected strong interspecific divergence within each haplotype group.

The second hypothesis, recent introgression at *Mpi *(and possibly *Ci*), but not at the other loci, is on balance more likely. The most convincing evidence of introgression in Panama is provided by the comparison between geographic populations within *melpomene*. At *Mpi *and *Ci*, *H. cydno *and *H. melpomene *share haplotype groups and have low divergence within Panama, while net divergences between races of *melpomene *are similar to those at other loci (Fig. 3, 4, Table 2). The allopatric French Guiana *H. melpomene melpomene *population acts as a control for divergence at these loci, and so the much lower net divergence between *H. melpomene rosina *and *H. cydno *in Panama (1/60 at *Mpi*, 1/3 at *Ci*) compared with divergence between either taxon and *H. melpomene *from French Guiana is clearly aberrant.

Additional evidence comes from nucleotide polymorphisms (Table 3). The ratio of fixed differences to shared polymorphisms is expected to increase with time since a split between populations. For *Co *and *Tpi*, our data are consistent with an ancient split between *H. melpomene *and *H. cydno*, and a much more recent split, or ongoing introgression between the two *H. melpomene *races. Only at *Mpi *are a large number of polymorphisms shared between *H. cydno *and *H. melpomene*, and then only in sympatric Panama populations. A *G *test of homogeneity in the frequencies of shared polymorphisms versus fixed site differences between *H. cydno *and *H. melpomene *in Panama strongly rejects the hypothesis that divergence and polymorphisms accumulated in the same proportions within each gene, as expected under neutral population divergence in total isolation (Table 3, central two columns, P << 0.001). Some loci show many fixed differences and few local polymorphisms; others show the opposite. This striking pattern can be explained most simply as a result of recent or ongoing interspecific gene flow in Panama only, leading to sharing of *Mpi *and possibly *Ci *polymorphisms between species, but not at other genes.

Finally, the IM analysis [18] suggests exchange of *Mpi *haplotypes between the two species in Panama, but no evidence of gene flow at other loci. Instead of being due to gene flow, IM suggests that polyphyly in the genealogies of *Ci *may be due to ancestral polymorphism inherited from a common ancestor, or possibly due to *Ci *haplotype introgression in the distant past. In only the most reduced dataset (IM reduced dataset 1) for *Ci *is there a relatively flat distribution of posterior probability for higher migration levels (amplified 10 × in Fig. 5 for clarity). In view of the stronger clustering of *Ci *haplotypes by species than at *Mpi*, a conclusion of little evidence for introgression at *Ci *seems warranted. The analysis therefore provides evidence for introgression only for *Mpi *haplotypes, where effective gene flow is about 1.5 × 10^-6 ^per generation (with a 95% credibility interval of 9 × 10^-7 ^– 4.50 × 10^-5^, Table 5).

Loci embedded in divergently selected genomic regions may be less likely to cross the species boundary than others [15]. Our data are consistent with linkage maps and knowledge of sterility between *H. melpomene *and *H. cydno*. Female hybrids between the two species are sterile [34], which should prevent mitochondrial transfer. (Female hybrids between Panama and French Guiana *H. melpomene *are also sterile, but in only one direction of the cross [40], so that mitochondrial transfer should still be possible between the geographic races, although here geography is probably the cause of divergence). *Tpi *may have a similar genealogy to that of *CoI*/*CoII *because the gene is syntenic with *Z*-linked female sterility in crosses [34]. *Ci *maps to linkage group 18 [37], which also contains an important locus causing fixed colour pattern differences between the two species. Introgression is thus likely to be inhibited in this locus by strong mimetic selection against intermediate or introgressed colour patterns [20,27,28]. In contrast, introgression of autosomal *Mpi *haplotypes (linkage group 3 [37]) between *H. cydno *and *H. melpomene *may occur more readily because it is unlinked to any loci known to be associated with sterility or other divergently selected traits.

The use of a color pattern 'toolbox' of switch genes shared between multiple species has been suggested as a means by which similar, homoplasious colour patterns spread throughout the *H. melpomene *– silvaniform clade of the genus *Heliconius *[24,41]. Many of these species hybridize in nature [5]. For example, *H. timareta, H. tristero and H. heurippa *from the Eastern Andes are considered separate species, although analyses of mitochondrial sequences place them with clades of *H. cydno *[23,24,30,31]. The taxa in this group are mostly Müllerian mimics of other *Heliconius*, and may have obtained their red-marked color patterns via hybridization and selective gene flow from local races of *H. melpomene *[30,31,41]. Occasional introgression may thus have allowed wholesale transfer of multilocus, ready-made mimetic adaptations. Our finding of introgressed autosomal haplotypes between two members of this subgroup provides molecular evidence for the possibility such a claim.

### Inferring history and species status from limited sequence data

The data presented here also illustrate the difficulties of reconstructing phylogeny of closely related species from DNA sequences. For this study, finished sequence was obtained from 30 individuals, and from 43–45 haplotypes for each nuclear gene. For each individual, cloning stages must be added so that some 10,000 extra bp DNA had to be sequenced per individual, or about 390,000 bp total to obtain reliable sequences for just four loci sequenced in every individual. Of the loci used, two (*Co *and *Tpi*) agree with prior morphologically-based understanding of species delimitation, showing no significant deviation from reciprocal monophyly. Two loci strongly reject the same phylogenetic interpretation, of which one (*Ci*) shows potential evidence for ancient gene flow and/or abundant ancestral polymorphism, and the other (*Mpi*) shows clear evidence for ongoing introgression between the species in sympatry and differentiation between geographic races. Still another locus tried (*Dopa decarboxylase *– *Ddc*, [see Additional file 1]) was inconclusive, although we hypothesise fixed differences and reciprocal monophyly for this locus   too, since *H. cydno *apparently lacks priming sites in *H. melpomene *and other related taxa. Previous conclusions that *H. cydno *is nested within Guiana and Panama *melpomene *clades [23] appear on current data to be incorrect, and may result from a lack of resolution due to the smaller *CoI*/*CoII *fragment used earlier. However, although mutual monophyly is not ruled out using the *Co *and *Tpi *fragments studied here, maximum likelihood genealogies of both genes suggest that species paraphyly is as, or more, likely. At the same time, convincing evidence for polyphyletic genealogies at *Ci *and *Mpi *show that introgression and ancestral polymorphisms within Panama may often mimic patterns concluded from the earlier mitochondrial data. Whether *H. melpomene *and *H. cydno *are mutually monophyletic is therefore no longer even a sensible question at the species level – the answer depends in which part of the genome we are interested.

Two loci examined here show no evidence for introgression (*Co *and *Tpi*). Of the two loci showing potential evidence for introgression via polyphyly, *Mpi *demonstrates fixed allelic differences, with no French Guiana alleles found within Panama clades, and little homoplasy between haplotypes; at *Ci*, in contrast, haplotypes are mixed freely among races of *melpomene*, as well as between species, and there is abundant evidence of recombination among haplotypes both within and between species. Although we did not expect such strong differences beween loci *a priori*, it is clear that there are many other differences in the evolution of the introns sampled at the four nuclear loci. The intron at *Mpi *is characterised by major splits between highly divergent haplotype groups maintained as polymorphisms (possibly as a result of balancing selection – see [24]), each haplotype group associated with particular indels, and showing little evidence of recombination. *Tpi *and *Ci *show many polymorphic indels, and strong evidence for recombination between haplotypes. Introns at *Ddc*, in contrast, [see Additional file 1] show strong sequence conservation, and are readily alignable even with distantly related species such as *H. himera *(a close relative of *H. erato*), while the highly divergent intronic sequences from *Tpi *or *Mpi *are impossible to align between *erato*-group and *melpomene*-group species of *Heliconius *[24]. *Ddc *also has few indels, and shows no evidence for recombination, even in its intronic sequences. A major finding of this study has been to demonstrate how idiosyncratically different genes can evolve. In this respect, our results are concordant with those obtained both for other sympatric, regularly hybridizing insect groups (e.g. *Anopheles gambiae *sensu lato [13,17] and the *Drosophila pseudoobscura *group [16,18]) and also in a largely allopatric radiation of three sibling species between which hybridization is unlikely and introgression not observed (*Drosophila simulans *group [42]). A 'species phylogeny' of closely related taxa such as these at best provides an artificial consensus of multiple conflicting genealogical patterns, rather than a meaningful representation of actual lineage diversification.

This study also highlights the difficulty of delimiting species on the basis of limited sequence information. In our populations, a short mtDNA barcode sequence could be used to assign individuals to clades each having fixed differences, and these clades could then be labelled as belonging either to *H. melpomene *or to *H. cydno *on the basis of already established biological, ecological and morphological traits. However, *a priori *attempts to define species boundaries on the basis of the mtDNA marker will normally fail: we might decide that all strongly supported branches of the mitochondrial tree in Fig. 1 were separate species (giving 5 taxa), or we might lump all *H. melpomene *with *H. cydno *into a single species. We would be most unlikely to hit on the biologically relevant current species classification, which reflects the fact that *H. cydno *and *H. melpomene *are sympatric, morphologically and ecologically differentiated, and show strong mate choice and unisexual hybrid sterility, while French Guiana haplotype groups form an evolutionary continuum with the Panamanian taxa across the northern coast of South America. Genealogical paraphyly or polyphyly at individual loci are simply not very good means of lumping taxa together as species [5,49].

Thus the recent tendency to split geographic forms into species based on fixed mitochondrial differences [49,51] but see [52] is inadequate without investigating a panel of nuclear genes as well. Oddly, this is similar to the criticism leveled by Zink [53] himself against the subspecies erected based on morphological characters: when such markers or characters are used to delimit taxa, the resulting taxon predicts little about the behaviour of the rest of the genome. Yet taxonomic inflation caused by elevation of populations to species level based on mtDNA data is surely a worse problem for taxonomy and conservation than difficulties at the subspecies level [54].

The considerable interlocus variation in mode of evolution and genealogical history demonstrates that large numbers of sequenced loci will be needed in order to investigate and identify phylogeny and species boundaries reliably, and even then will provide only a consensus of genealogies, because a 'true' species phylogeny is not possible when genealogies conflict. In particular, taxon delimitation based on mitochondrial sequences alone will give little insight into the behaviour of nuclear genes. It could be argued that *Heliconius *is a special case, but very appreciable per species rates of hybridization are found both in *Heliconius *butterflies and in many bird groups currently undergoing mtDNA-based species delimitation and taxonomic inflation [5]. We argue that morphological, ecological, and behavioural data, coupled with geographical distribution data (particularly sympatry), will remain superior to DNA sequences for species delimitation [52], at least until larger numbers of loci can be readily analysed using a truly 'genealogical genomics' approach.

## Conclusion

This work adds to a small but growing body of DNA sequence evidence showing that genetic material may pass regularly between closely related animal species in nature, millions of generations after speciation [16,20]. This work supports the hypothesis that introgressive hybridisation could make a significant contribution to adaptive evolution in *Heliconius *and in animals generally. If it is generally true that closely related, non-sibling, ecologically distinct animal species are often permeable to introgression, the nature of such species, their ecology, causes of speciation [1,16], phylogeny reconstruction [43], as well as conservation issues concerning hybridizing populations [44] all require re-evaluation. In particular, phylogenetic reconstruction and diagnostic tests for species status employing single genes must be used with great caution [45-48]. Species boundaries in radiating groups, even in 'normal' continental species such as *Heliconius*, appear to remain porous long after divergence.

## Methods

### Sampling methods and DNA extraction

Thirty wild butterflies were sampled (28 males and 2 females), 10 each of *H. melpomene rosina *from Panama, *H. melpomene melpomene *from French Guiana, and *H. cydno chioneus *from Panama. Sequences from *H. numata *were used as outgroups. (Genealogies were also checked using other related *Heliconius*, as well as *H. himera *as outgroups; data not shown). Butterflies were collected in the field, and preserved in liquid nitrogen. These samples are stored at -70°C at the Smithsonian Tropical Research Institute in Panama. From each individual, 1/3 of a thorax was ground in liquid nitrogen, and genomic DNA was extracted using the standard phenol-chloroform method [58].

### Loci and primers

A region of mtDNA spanning the 3' end of *CoI*, *leucine-tRNA *(*tRNA-leu*), and *CoII *was selected as a suitable mitochondrial region following work by Beltrán *et al*. [22,24]. This mitochondrial region has been used in many insect studies, and a shorter region of *CoII *included within our study was used in pioneering molecular phylogenetic studies of the genus *Heliconius *[23,59]. *Tpi *is an important enzyme in carbohydrate metabolism encoded by a sex-linked nuclear gene in most Lepidoptera [60]. The region amplified spans a single intron in *Heliconius *[24], is inherited in a Mendelian manner and is sex-linked in *Heliconius *[27,34,37,40,61] and very likely many other Lepidoptera (e.g. *Ostrinia *– [62]; and *Bombyx *– see GenBank accession AY734490). *Mpi *also spans a single intron and is encoded by an autosomal gene. The protein product is highly polymorphic in Lepidoptera, including *Heliconius *[63-66]. Two other loci, *Ci *and *Ddc *were developed because of their possible involvement in wing pigmentation genetics of butterflies [61]. *Ci *is a transcription factor serving to activate the transcription of *wingless *[70], and involved in wing-patterning in some butterflies [71]; the fragment studied here spanned two introns. *Ddc *is involved in the melanin pathway in insects, where it catalyses the conversion of dopa to dopamine [67-69]; the fragment studied here spanned two introns.

Primers and details of methods for all the loci have been described earlier [24,37,61,72]; further details are given in [Additional file 1].

Because of the many insertions and deletions in the intron-spanning sequences studied here, direct sequencing produced ambiguous base calls in heterozygotes. We therefore separated the two alleles of nuclear loci by cloning prior to sequencing. Products amplified from genomic DNA were run in a low-melting point agarose gel (as for mtDNA) and the bands excised and dissolved in agarase. The products were cloned to obtain the sequence for each allele, using pGEM ^®^-T Easy Vector System II (Promega). Five or more clones per individual were selected; re-amplified, and again purified on an agarose gel. Positive bands were excised and dissolved using agarase.

### Purification, sequencing and allele editing

Templates from all loci were cycle sequenced using primers and methods already published [24,37]. PCR will generate *Taq *errors of amplification, and these errors can be 'fixed' when extra steps of cloning and PCR are added [73-75]. To correct these errors, we sequenced a minimum of five clones per individual. These were aligned and sorted into haplotype classes ('A' and 'B' where shown to be heterozygous), and the consensus sequence was deduced by assuming that single-base *Taq *error was likely to occur only once. During this procedure, we found one individual (CP569) for which one clone had a recombinant *Tpi *allele, which matched the A allele for part of its length and then the B allele for the rest, clearly resulting from *Taq*-induced recombination during the initial PCR stage. In no other case could sequenced clones from each individual be interpreted as belonging to more than two alleles of *Mpi*, *Tpi*, *Ci *per individual, giving further evidence against duplicate loci.

### Testing for duplicate loci, pseudogenes and other anomalous sequences

To test for the presence of pseudogenes and other duplicates, we checked our sequences against those already obtained for related species, and for the stop codons expected in pseudogene sequences. In no case did we detect anomalies. At *Mpi*, there were major divergences in sequence within species, populations and even individuals (as had already been discovered in *Heliconius*[24]), and as the evidence for introgression was strongest at this locus, it was important to check for the possibility of duplicate loci. We screened for heterozygosity using Temporal Temperature Gradient gel Electrophoresis (TTGE) [76]. *Mpi *clone were run using 8 μl of double stranded PCR product using the BioRad TTGE 'Dcode' system. Gels contained 8% acrylamide and 1.75 M tris acetate EDTA buffer (TAE), and were run from 46 – 53°C at a temperature ramp of 1°C per hour. In no case were more than two alleles observed in any individual. Several broods were also tested to verify Mendelian segregation using RFLP polymorphisms derived from the sequence information, and all autosomal and sex-linked loci behaved as expected for single loci in broods mapped using AFLP markers [34,37]. Haplotypes therefore segregate in the expected Mendelian fashion and were inherited in complete linkage with the *Mpi *allozyme locus in broods of *H. erato *and *himera *[61]. We conclude that *Mpi *behaves as expected for a single-copy locus, in spite of its high intraspecific variability, which may be related to hybridization and introgression (see below).

### Sequence alignment and phylogenetic/genealogical analysis

Chromatograms for all genes were edited, base calls were checked and aligned manually. Complete original alignments are given in [Additional file 2]. All single base polymorphisms occurring in only one or two individuals were rechecked against chromatograms to ensure they were correctly read prior to phylogenetic analysis. All sequences were translated to check for reading-frame errors and stop codons. The new haplotype sequences studied here are deposited under GenBank accessions AF512970-AF512993 (*Co*), AF516210-AF516255 (*Mpi*), AF545437-AF545469 (*Tpi*), AY429261-AY429304 (*Ci*), and complete alignments are given in [Additional file 2]. Sequences were verified by aligning against *Heliconius *cDNA sequences, or other Lepidoptera or *Drosophila *sequences for the same gene. An unusual insertion and deletion in *Ci *consisting of an approx 270 bp sequence was found in three clones (*melpomene rosina *MP 545A and MP 545B, and *cydno *CP 809A). This indel aligned well between these three clones, but was unalignable with any other sequence, including those from other *Heliconius*. The region was clearly homoplasious with the rest of the genealogy and was deleted prior to analysis. Unalignable sequences are a widespread problem in molecular evolutionary biology [77], and their deletion can lead to a loss of information. However, with the insertion included, the topology was nearly identical, the main difference being the extremely long branch lengths and similar sequences of the three problem haplotypes tended to cause them to group together. Net divergence estimates and shared polymorphism counts were also performed on the data set with this unalignable region excluded. Apart from these sequence fragments, the entire sequences were used to obtain an estimate of genealogy.

Phylogenetic analyses were performed with PAUP* version 4.0b10 [78]. Models of sequence evolution were compared by means of likelihood ratio tests using ModelTest 3.04 [79]. PAUP* was then used to search for the maximum likelihood (ML) tree, based on the best fit model and parameter estimates given by ModelTest (Table 1) and using a heuristic search with tree bisection reconnection (TBR). Confidence in different hypotheses (e.g. constrained to mutual monophyly versus maximum likelihood) was tested using the bootstrapped Shimodaira-Hasegawa likelihood-ratio test (SH test [80]) as implemented by PAUP*.

For comparison, maximum parsimony trees were also obtained using a heuristic search with TBR branch swapping. Confidence in each node was assessed by bootstrapping (10000 replicates also with TBR branch swapping). Because we were most interested in branch support, we did not analyse the data using Bayesian methods, because the very high branch support that this method produces has come under suspicion, and may be due to great sensitivity of Bayesian phylogenetic analysis to the form of the prior distribution assumed [81]. In addition to phylogenetic analysis, the data were analyzed to estimate a range of population genetic parameters. Polymorphism and divergence estimates (Table 2, 3) were calculated using the SITES program [82,83].

To estimate the importance of introgression, we initially attempted to use the Wakeley-Hey (WH) algorithm [14,83] to test the null hypothesis of equal rates of accumulation of divergence and polymorphisms, but the simulation-based computer implementation (WH) failed to complete. This appears to be a common situation for data of this kind where relatively few loci, highly heterogeneous for levels of divergence, are used to estimate ancestral population sizes [14,17]. In addition, the SITES program and the WH test are not very appropriate because they assume an infinite sites model, under which a single site cannot be substituted twice in the same genealogy. However, polymorphisms with three bases are quite common in all of the rapidly evolving sequences studied here. Another problem was that any sites with missing data or spanned by indels in one or more aligned sequences are ignored. Our intron data often have indels, so that, summed over all individuals, much of the sequence may be spanned by one or more indels leading to a complete loss of information in SITES analyses.

We therefore analysed the data using the Isolation-Migration (IM) method [18]. The program employs a Metropolis-Coupled Monte-Carlo Markov-Chain (MCMCMC) algorithm for Bayesian estimation of genealogical parameters related to mutation and introgression in a single pair of species. The programme has recently been upgraded to deal with bugs which affected the HKY mutation model used here (November 2005, Hey pers. comm.); all analyses were re-run with the new executable files. Like SITES, the program still ignores DNA sites for which any sequence has missing data, and the program also assumes no recombination within each sequenced locus. SITES revealed a certain amount of 'recombinant' sequences, particularly at the *Ci *locus, so IM could give misleading results using our data. However, SITES uses a four-gamete test for recombination between individual sites, which is valid only under the infinite sites mutation model, and so gives a criterion likely to be much too strict for more realistic, HKY-type models of evolution, since repeated changes at the same site were observed. A better clue to recombination is the overall pattern of multi-site sequences, and genealogical homoplasy of indels (Fig. 2, 3, 4), which may be less likely to recur via mutation than single base pair changes (particularly transitions under HKY and more complex models). We also tested for recombination using a model-neutral test based on a bootstrapped correlation of linkage disequilibrium (*R*^2^) with physical distance [38]. Following Hey & Nielsen [18, J. Hey pers. comm. 2005], for the IM analysis, therefore, the data were pared down, firstly to remove any indel information not analysable by IM, while maximizing the sequence data (the basic IM dataset), and then subsampled to remove clearly recombinant regions, by sampling from the 5' region of each gene until a probable recombinant pattern is observed (reduced dataset 1). Because the 5' apparently unrecombined region of the *Ci *locus was very short, we also used a different subset containing instead the longer 3' unrecombined region of *Ci *(reduced dataset 2). 'Inheritance scalars' (per locus constant effective population sizes relative to those for an autosomal locus) were set at 0.25 for *Co*, 0.75 for the sex-linked *Tpi*, and 1.00 for the other loci. All loci were used in each run to estimate individual species and ancestral population sizes θ = 4 *N*μ, along with parameters time of divergence (*t*), relative mutation/substitution rates rates (μ), and per locus bidirectional gene flow (*m*). These parameters were calibrated to a molecular clock to obtain parameters per base pair and per generation via Brower's [32] estimate of insect mitochondrial divergence of 2.3% per million years (i.e. a neutral substitution rate, μ, of 1.15% per million years), and with four generations per year assumed in *Heliconius*. Neutral mutation (substitution rates) and migration rates (*m*) were allowed to vary between loci. However, to reduce the numbers of parameters, introgression was assumed symmetrical within each locus (using the terminology of IM, *m*1=*m*2, i.e. option -j56). After optimizing parameter search limits using initial runs, each of the three datasets were run for at least 30 million steps after burn-in under the HKY model in IM using 5 chains per set, with linear heating increment parameters, *h *of 0.033, and a discarded burn-in of 200,000 steps. Actual run durations after burn in were 35,729,000 steps for the IM dataset, 49,769,000 steps for IM reduced dataset 1, and 54,460,000 steps for IM reduced dataset 2. The three IM datasets used have been provided in [Additional file 2].

## Authors' contributions

VB and MB carried out molecular laboratory work, with the help of CJ, WOM, and EB. VB and JM analysed the data. All authors participated in the conception and design of the study, collection of specimens, writing, and approval of the final manuscript.

## Supplementary Material

Additional File 1 **Laboratory protocols and the *Dopa decarboxylase *locus**. Laboratory protocols for loci sequenced, and details of the *Dopa decarboxylase *locus. Click here for file

Additional File 2 **Complete alignment data**. Aligned sequences for phylogenetic analysis and IM analysis. Click here for file

## Figures and Tables

**Table 1:** Summary statistics and maximum likelihood model parameters for gene regions. Ten individuals were sampled from each of the three populations, and the same individuals were sequenced for all genes. For nuclear loci, one or two sequences per individual was obtained via cloning of diploid genes. To investigate introgression, paired datasets for sympatric *H. cydno *and *H. melpomene *only from Panama (10 individuals each) were analysed using the IM Bayesian algorithm [18]; these are the IM dataset, and IM reduced datasets 1 and 2 – the latter have been purged of presumably recombinant sequences by deleting some individuals and by sampling from 5' or 3' regions of each gene (see methods). Complete alignment data in FASTA format and IM data file format are given in [Additional file 2 ].

	*CoI/II*	*Tpi*	*Mpi*	*Ci*
No. haplotypes sequenced overall	30	45	45	43
No. haplotypes IM dataset	20	31	27	12
No. haplotypes IM reduced dataset 1	20	31	26	12
No. haplotypes IM reduced dataset 2	20	31	26	12

No. of base pairs of finished sequence	1572	569	453	840
IM dataset	1571	419	315	660
IM reduced dataset 1	1571	245 (5')	315	95 (5')
IM reduced dataset 2	1571	245 (5')	315	495 (3')

Total no. variable sites	92	62	70	186
Variable sites in non-coding region	1	55	66	167
No. variable sites in coding region	91	7	4	19
No. variable sites by codon position (1^st^/2^nd^/3^rd^)	9/0/82	2/0/5	1/0/3	2/2/15
Recombination in IM dataset? (MP, MG, CP, MP+CP)	- - - -	+ - + -	- + - -	+ + + +
Maximum likelihood model selected	TrN+I+G	TrN+G	HKY+G	TrN+I+G
Proportion of invariable sites (I)	0.80	-	-	0.43
Gamma shape parameter (G)	0.90	0.22	0.44	0.75
Base composition				
A	0.34	0.36	0.37	0.35
C	0.13	0.14	0.13	0.14
G	0.12	0.18	0.11	0.16
T	0.41	0.32	0.39	0.35
Relative substitution rates				
A-C	1	1	1	1
A-G	13.58	1.43	1.50	1.54
A-T	1	1	1	1
C-G	1	1	1	1
C-T	35.7	3.6	1.50	2.95
G-T	1	1	1	1

**Table 2:** Uncorrected average pairwise divergence per base pair on and above diagonal, and net pairwise divergence (with intrapopulation average divergence subtracted) below diagonal for the four loci studied.

			*H. melpomene*		*H. cydno*
			Panama	French Guiana	Panama

*CoI and CoII*	*H. melpomene*	Panama	0.0092	0.0146	0.0376
		French Guiana	0.0038	0.0125	0.0345
	*H. cydno*	Panama	0.0299	0.0251	0.0063
					
*Tpi*	*H. melpomene*	Panama	0.0079	0.0218	0.0283
		French Guiana	0.0133	0.0078	0.0402
	*H. cydno*	Panama	0.0136	0.0255	0.0212
					
*Mpi*	*H. melpomene*	Panama	0.0394	0.0449	0.0454
		French Guiana	0.0191	0.0109	0.0515
	*H. cydno*	Panama	0.0003	0.0201	0.0497
					
*Ci*	*H. melpomene*	Panama	0.0561	0.0361	0.0727
		French Guiana	0.0372	0.0185	0.0507
	*H. cydno*	Panama	0.0073	0.0290	0.0473

**Table 3:** Numbers of shared single nucleotide polymorphisms and fixed differences.

Locus	MP/MG		MP/CP		CP/MG	
	Shared polymorphisms	Fixed differences	Shared polymorphisms	Fixed differences	Shared polymorphisms	Fixed differences

*CoI*/*II*	38	0	5	32	5	27
*Tpi*	4	0	1	3	1	3
*Mpi*	4	0	37	0	4	1
*Ci*	40	0	38	0	39	0

**Table 4:** Genealogical parameter estimates from Bayesian analysis of IM uncorrected dataset

		95% credibility interval	
	Estimate	Lower limit	Upper limit

*μ*_*Co*_	2.88 × 10^-9^	1.38 × 10^-9^	5.50 × 10^-9^
*μ*_*Tpi*_	1.33 × 10^-8^	7.63 × 10^-9^	2.21 × 10^-8^
*μ*_*Mpi*_	1.41 × 10^-8^	8.25 × 10^-9^	2.60 × 10^-8^
*μ*_*Ci*_	9.67 × 10^-9^	7.44 × 10^-9^	1.98 × 10^-8^
			
*θ*_*mel*_	0.00306	0.00192	0.00539
*θ*_*cyd*_	0.02187	0.01404	0.05051
*θ*_*A*_	~0*	~ 0	>0.61840*
			
*t*	2,073,000	1,137,000	>5,405,000*
			
*m*_*Co*_	~0	~0	6.44 × 10^-7^
*m*_*Tpi*_	~0	~0	2.29 × 10^-6^
*m*_*Mpi*_	1.64 × 10^-6^	1.10 × 10^-6^	6.37 × 10^-5 ^*
*m*_*Ci*_	~0	~0	8.59 × 10^-5^

*μ*_*L *_= mutation rate per base pair per generation for each locus *L*.

*θ*_*sp *_= combined parameter 4 *N*_*e*_*μ*_*Co *_of species '*sp*', or their ancestor '*A*', relative to the mitochondrial mutation rate *μ*_*Co*_

*N*_*e *_represents the effective population size.

*t *= time of divergence in years.

*m*_*L *_= locus-specific migration rates per generation for each locus *L*.

* = unreliable estimate or limit due to flat or incomplete posterior probability distribution sampled.

~0 = effectively zero, although the lowest 'bin' does not actually include zero.

**Table 5:** Genealogical parameter estimates from Bayesian analysis of IM reduced dataset 1

		95% credibility interval	
	Estimate	Lower limit	Upper limit

*μ*_*Co*_	2.88 × 10^-9^	1.46 × 10^-9^	5.43 × 10^-9^
*μ*_*Tpi*_	9.76 × 10^-9^	5.47 × 10^-9^	1.72 × 10^-8^
*μ*_*Mpi*_	9.87 × 10^-9^	5.68 × 10^-9^	1.79 × 10^-8^
*μ*_*Ci*_	1.01 × 10^-8^	7.64 × 10^-10^	2.19 × 10^-8^
			
*θ*_*mel*_	0.00207	0.00127	0.00391
*θ*_*cyd*_	0.00875	0.00538	0.01996
*θ*_*A*_	~0*	~0	>0.43377*
			
*t*	2,804,000	750,000	>5,405,000*
			
*m*_*Co*_	~0	~0	1.39 × 10^-6^
*m*_*Tpi*_	~0	~0	1.24 × 10^-6^
*m*_*Mpi*_	1.70 × 10^-6^	1.00 × 10^-6^	>4.44 × 10^-5 ^*
*m*_*Ci*_	~0	~0	>1.40 × 10^-5 ^*

Legend as for Table 4

**Table 6:** Genealogical parameter estimates from Bayesian analysis of IM reduced dataset 2

		95% credibility interval	
	Estimate	Lower limit	Upper limit

*μ*_*Co*_	2.88 × 10^-9^	1.48 × 10^-9^	5.43 × 10^-9^
*μ*_*Tpi*_	9.90 × 10^-9^	5.54 × 10^-9^	1.70 × 10^-8^
*μ*_*Mpi*_	1.00 × 10^-8^	5.84 × 10^-9^	1.84 × 10^-8^
*μ*_*Ci*_	3.37 × 10^-9^	6.08 × 10^-10^	7.21 × 10^-9^
			
*θ*_*mel*_	0.00227	0.00142	0.00430
*θ*_*cyd*_	0.00996	0.00646	0.02263
*θ*_*A*_	~0*	~0	>0.61967*
			
*t*	1,962,000	922,000	>5,405,000*
			
*m*_*Co*_	~0	~0	1.19 × 10^-6^
*m*_*Tpi*_	~0	~0	1.04 × 10^-6^
*m*_*Mpi*_	1.54 × 10^-6 ^*	8.75 × 10^-7^	>4.48 × 10^-5 ^*
*m*_*Ci*_	~0	~0	2.31 × 10^-5^

Legend as for Table 4

## References

[B1] Arnold ML (1997). Natural Hybridization and Evolution.

[B2] Rieseberg LH (1997). Hybrid origins of plant species. Ann Rev Ecol Syst.

[B3] Dowling TE, Secor CL (1997). The role of hybridization and introgression in the diversification of animals. Ann Rev Ecol Syst.

[B4] Coyne JA, Orr HA (2004). Speciation.

[B5] Mallet J (2005). Hybridization as an invasion of the genome. Trends Ecol Evol.

[B6] Grant PR, Grant BR (1992). Hybridization of bird species. Science.

[B7] Guillaumin M, Descimon H, Bocquet C, Génermont J, Lamotte M (1976). La notion d'espèce chez les lépidoptères. Les Problèmes de l'Espèce dans le Règne Animal.

[B8] della Torre A, Merzagora L, Powell JR, Coluzzi M (1997). Selective introgression of paracentric inversions between two sibling species of the *Anopheles gambiae *complex. Genetics.

[B9] Rieseberg LH, Whitton J, Gardner K (1999). Hybrid zones and the genetic architecture of a barrier to gene flow between two sunflower species. Genetics.

[B10] Walton C, Handley JM, Collins FH, Baimai V, Harbach RE, Deesin V, Butlin RK (2001). Genetic population structure and introgression in *Anopheles dirus *mosquitoes in South-east Asia. Molec Ecol.

[B11] Wilding CS, Butlin RK, Grahame J (2001). Differential gene exchange between parapatric morphs of *Littorina saxatilis *detected using AFLP markers. J Evol Biol.

[B12] Emelianov I, Marec F, Mallet J (2004). Genomic evidence for divergence with gene flow in host races of the larch budmoth. Proc Roy Soc Lond B.

[B13] Turner TL, Hahn MW, Nuzhdin SV (2005). Genomic islands of speciation in *Anopheles gambiae*. PLoS Biol.

[B14] Wang RL, Wakeley J, Hey J (1997). Gene flow and natural selection in the origin of *Drosophila pseudoobscura *and close relatives. Genetics.

[B15] Ting C-T, Tsaur S-C, Wu C-I (2000). The phylogeny of closely related species as revealed by the genealogy of a speciation gene, *Odysseus*. Proc Natl Acad Sci USA.

[B16] Machado CA, Kliman RM, Markert JA, Hey J (2002). Inferring the history of speciation from multilocus DNA sequence data: the case of *Drosophila pseudoobscura *and its close relatives. Molec Biol Evol.

[B17] Besansky NJ, Krzywinski J, Lehmann T, Simard F, Kern M, Mukabayire O, Fontenille D, Touré Y, Sagnon N'F (2003). Semipermeable species boundaries between *Anopheles gambiae *and *Anopheles arabiensis*: evidence from multilocus DNA sequence variation. Proc Natl Acad Sci USA.

[B18] Hey J, Nielsen R (2004). Multilocus methods for estimating population sizes, migration rates and divergence times, with applications to the divergence of *Drosophila pseudoobscura *and *D. persimilis*. Genetics.

[B19] Brown KS (1981). The biology of *Heliconius *and related genera. Ann Rev Entomol.

[B20] Turner JRG (1981). Adaptation and evolution in *Heliconius *: a defense of neo-Darwinism. Ann Rev Ecol Syst.

[B21] Mallet J, McMillan WO, Jiggins CD, Howard DJ, Berlocher SH (1998). Mimicry and warning color at the boundary between races and species. Endless Forms: Species and Speciation.

[B22] Beltrán M, Jiggins CD, Brower AVZ, Bermingham E, Mallet J (2006). Do pollen feeding and pupal-mating have a single origin in *Heliconius *? Inferences from multilocus sequence data. Biol J Linn Soc.

[B23] Brower AVZ (1996). Parallel race formation and the evolution of mimicry in *Heliconius *butterflies: a phylogenetic hypothesis from mitochondrial DNA sequences. Evolution.

[B24] Beltrán MS, Jiggins CD, Bull V, Linares M, Mallet J, McMillan WO, Bermingham E (2002). Phylogenetic discordance at the species boundary: comparative gene genealogies among rapidly radiating *Heliconius *butterflies. Molec Biol Evol.

[B25] Smiley JT (1978). Plant chemistry and the evolution of host specificity: new evidence from *Heliconius *and *Passiflora*. Science.

[B26] Estrada C, Jiggins CD (2002). Patterns of pollen feeding and habitat preference among *Heliconius *species. Ecol Entomol.

[B27] Jiggins CD, Naisbit RE, Coe RL, Mallet J (2001). Reproductive isolation caused by colour pattern mimicry. Nature.

[B28] Kapan D (2001). Three-butterfly system provides a field test of Müllerian mimicry. Nature.

[B29] Brower AVZ (1996). A new mimetic species of *Heliconius *(Lepidoptera: Nymphalidae), from southeastern Colombia, revealed by cladistic analysis of mitochondrial DNA sequences. Zool J Linn Soc.

[B30] Mavárez J, Salazar C, Bermingham E, Salcedo C, Jiggins CD, Linares M (2006). Hybrid speciation in *Heliconius *butterflies. Nature.

[B31] Salazar C, Jiggins CD, Linares M (2006). Multilocus genetic evidence for hybrid speciation in a neotropical butterfly. PLoS Biol.

[B32] Brower AVZ (1994). Rapid morphological radiation and convergence among races of the butterfly *Heliconius erato *inferred from patterns of mitochondrial DNA evolution. Proc Natl Acad Sci USA.

[B33] Naisbit RE, Jiggins CD, Mallet J (2001). Disruptive sexual selection against hybrids contributes to speciation between *Heliconiuscydno *and *H. melpomene*. Proc Roy Soc Lond B.

[B34] Naisbit RE, Jiggins CD, Linares M, Mallet J (2002). Hybrid sterility, Haldane's rule, and speciation *Heliconius cydno *and *H. melpomene*. Genetics.

[B35] Naisbit RE, Jiggins CD, Mallet J (2003). Mimicry: developmental genes that contribute to speciation. Evol Devel.

[B36] Pacheco NM, Congdon BC, Friesen VL (2002). The utility of nuclear introns for investigating hybridization and genetic introgression: a case study involving *Brachyramphus *murrelets. Conserv Genet.

[B37] Jiggins CD, Mavarez J, Beltrán M, McMillan WO, Johnston JS, Bermingham E (2005). A genetic linkage map of the mimetic butterfly, *Heliconius melpomene*. Genetics.

[B38] Piganeau G, Gardner M, Eyre-Walker A (2004). A broad survey of recombination in animal mitochondria. Molec Biol Evol.

[B39] Flanagan NS, Tobler A, Davison A, Pybus OG, Kapan DD, Planas S, Linares M, Heckel D, McMillan WO (2004). The historical demography of Müllerian mimicry in the neotropical *Heliconius *butterflies. Proc Natl Acad Sci USA.

[B40] Jiggins CD, Linares M, Naisbit RE, Salazar C, Yang Z, Mallet J (2001). Sex-linked hybrid sterility in a butterfly. Evolution.

[B41] Gilbert LE, Boggs CL, Watt WB, Ehrlich PR (2003). Adaptive novelty through introgression in *Heliconius *wing patterns: evidence for shared genetic "tool box" from synthetic hybrid zones and a theory of diversification. Ecology and Evolution Taking Flight: Butterflies as Model Systems.

[B42] Kliman RM, Andolfatto P, Coyne JA, Depaulis F, Kreitman M, Berry AJ, McCarter J, Wakeley J, Hey J (2000). The population genetics of the origin and divergence of the *Drosophila simulans *complex species. Genetics.

[B43] Sang T, Zhong Y (2000). Testing hybridization hypotheses based on incongruent gene trees. Syst Biol.

[B44] Allendorf FW, Leary RF, Spruell P, Wenburg JK (2001). The problems with hybrids: setting conservation guidelines. Trends Ecol Evol.

[B45] Schluter D (2000). The Ecology of Adaptive Radiation.

[B46] Verheyen E, Salzburger W, Snoeks J, Meyer A (2003). Origin of the superflock of cichlid fishes from Lake Victoria, East Africa. Science.

[B47] Seehausen O (2003). Hybridization and adaptive radiation. Trends Ecol Evol.

[B48] Feder JL, Berlocher SH, Roethele JB, Dambrowski H, Smith JJ, Perry WL, Gavrolic V, Filchak KE, Rull J, Aluja M (2003). Allopatric genetic origins for sympatric speciation in *Rhagoletis*. Proc Natl Acad Sci USA.

[B49] Hudson RR, Coyne JA (2002). Mathematical consequences of the genealogical species concept. Evolution.

[B50] Zink RM, McKitrick MC (1995). The debate over species concepts and its implications for ornithology. Auk.

[B51] Zink RM (1996). Bird species diversity. Nature.

[B52] Remsen JV (2005). Pattern, process and rigor meet classification. Auk.

[B53] Zink RM (2004). The role of subspecies in obscuring avian diversity and misleading conservation policy. Proc Roy Soc Lond B.

[B54] Isaac NJB, Mallet J, Mace GM (2004). Taxonomic inflation: its influence on macroecology and conservation. Trends Ecol Evol.

[B55] Hudson RR, Turelli M (2003). Stochasticity overrules the "three-times rule": genetic drift, genetic draft, and coalescence times for nuclear loci versus mitochondrial DNA. Evolution.

[B56] Hebert PDN, Cywinska A, Ball SL, deWaard JR (2003). Biological identifications through DNA barcodes. Proc Roy Soc Lond B.

[B57] Hebert PDN, Penton EH, Burns JM, Janzen DH, Hallwachs W (2004). Ten species in one: DNA barcoding reveals cryptic species in the neotropical skipper butterfly *Astraptes fulgerator*. Proc Natl Acad Sci USA.

[B58] Harrison RG, Rand DM, Wheeler WC (1987). Mitochondrial DNA variation in field crickets across a narrow hybrid zone. Molec Biol Evol.

[B59] Brower AVZ, Egan MG (1997). Cladistic analysis of *Heliconius *butterflies and relatives (Nymphalidae: Heliconiiti): a revised phylogenetic position for *Eueides *based on sequences from mtDNA and a nuclear gene. Proc Roy Soc Lond B.

[B60] Logsden JM, Tyshenko MG, Dixon C, Javari JD, Walker VK, Palmer JD (1995). Seven newly discovered intron positions in the triose phosphate isomerase gene: evidence for the introns-late theory. Proc Natl Acad Sci USA.

[B61] Tobler A, Kapan D, Flanagan NS, Gonzalez C, Peterson E, Jiggins CD, Johnston JS, Heckel DG, McMillan WO (2005). First-generation linkage map of the warningly colored butterfly *Heliconius erato*. Heredity.

[B62] Glover T, Campbell M, Robbins P, Roelofs W (1990). Sex-linked control of sex pheromone behavioral responses in European corn borer moths (*Ostrinia nubilialis*) confirmed with *Tpi *marker gene. Arch Insect Biochem Physiol.

[B63] Turner JRG, Johnson MS, Eanes WF (1979). Contrasted modes of evolution in the same genome: allozymes and adaptive change in *Heliconius*. Proc Natl Acad Sci USA.

[B64] Jiggins CD, McMillan WO, King P, Mallet J (1997). The maintenance of species differences across a *Heliconius *hybrid zone. Heredity.

[B65] Raijmann LEL, Van Ginkel WE, Heckel DG, Menken SBJ (1997). Inheritance and linkage of isozymes in *Yponomeuta padellus*. Heredity.

[B66] Beltrán M (1999). Evidencia genética (alozimas) para evaluar el posible orígen híbrido de Heliconius heurippa (Lepidoptera: Nymphalidae).

[B67] Koch PB, Keys DN, Rocheleau T, Aronstein K, Blackburn M, Carroll SB, ffrench-Constant RH (1998). Regulation of dopa decarboxylase expression during colour pattern formation in wild-type and melanic swallowtail butterflies. Development.

[B68] Fang QQ, Mitchell A, Regier JC, Mitter C, Friedlander TP, Poole RW (2000). Phylogenetic utility of the nuclear gene *dopa decarboxylase *in noctuoid moths (Insecta: Lepidoptera: Noctuoidea). Molec Phylog Evol.

[B69] McMillan WO, Monteiro A, Kapan D (2001). Development and evolution on the wing. Trends Ecol Evol.

[B70] Motzny CK, Holmgren R (1995). The *Drosophila cubitus interruptus *protein and its role in the *wingless *and *hedgehog *signal transduction pathways. Mech Dev.

[B71] Beldade P, Brakefield PM (2002). The genetics and evo-devo of butterfly wing patterns. Nature Reviews Genetics.

[B72] Simon C, Frati F, Beckenbach A, Crespi B, Liu H, Flook P (1994). Evolution, weighting, and phylogenetic usefulness of mitochondrial genes with a compilation of conserved PCR primers. Ann Entomol Soc Amer.

[B73] Wang GCY, Wang Y (1997). Frequency of formation of chimeric molecules is a consequence of PCR coamplification of 16S rRNA genes from mixed bacterial genomes. Appl Environ Microbiol.

[B74] Bracho MA, Moya A, Barrio E (1998). Contribution of *Taq *polymerase-induced errors to the estimation of RNA virus diversity. J Gen Virol.

[B75] Kobayashi NK, Tamura K, Aotsuka T (1999). PCR error and molecular population genetics. Biochem Genet.

[B76] Orita M, Iwahana H, Kanazawa H, Hayashi K, Sekita T (1989). Detection of polymorphisms of human DNA by gel electrophoresis as single strand conformation polymorphism. Proc Natl Acad Sci USA.

[B77] Lee MSY (2001). Unalignable sequences and molecular evolution. Trends Ecol Evol.

[B78] Swofford DL (2000). PAUP*: Phylogenetic Analysis Using Parsiomony (*and Other Methods.

[B79] Posada NM, Crandall KA (1998). *MODELTEST *: testing the model of DNA substitution. Bioinformatics.

[B80] Shimodaira H, Hasegawa M (1999). Multiple comparisons of log-likelihoods with applications to phylogenetic inference. Molec Biol Evol.

[B81] Yang Z, Rannala B (2005). Branch-length prior influences Bayesian posterior   probability of phylogeny. Syst Biol.

[B82] Hey J, Wakeley J (1997). A coalescent estimator of the population recombination rate. Genetics.

[B83] Wakeley J, Hey J (1997). Estimating ancestral population parameters. Genetics.

