# Supplementary material for: Polyphyly and gene flow between non-sibling Heliconius species
Source: BMC Biol. 2006 Apr 21;4:11. doi: 10.1186/1741-7007-4-11 (PMC1481601; doi:10.1186/1741-7007-4-11)
Supplement: Additional File 1 — Laboratory protocols and the Dopa decarboxylase locus. Laboratory protocols for loci sequenced, and details of the Dopa decarboxylase locus. [file 1741-7007-4-11-S1.pdf]

Additional File 1 for Vanessa Bull et al. (2006). – Laboratory protocols for loci sequenced, and for the *Dopa decarboxylase* gene

Laboratory protocols

The mitochondrial region was amplified from genomic DNA in two parts using primers Jerry and Pat for *CoI* and George III and Imelda for *CoII* [24,72]. The primers for *Tpi* spanned intron 3 of the gene and were situated in exon 3 and 4, while *Mpi* primers amplified intron 3 and were situated in exons 3 and 4 [24]. For *Ci*, primers were designed by searching for conserved regions in cDNA sequences from *Heliconius himera*, *Junonia coenia* and *Drosophila melanogaster*. These primers amplify a region homologous to positions 1662-1860 in *Junonia coenia* (GenBank AF091245), which spans two introns (1 and 2) in *Heliconius*. We also tested another locus for this study, *Dopa decarboxylase* (*Ddc*), for which sequence data was generated, although the locus was rejected due to the possibility of a duplicate locus, as outlined below. *Ddc* primers were designed from *H. himera*, *Manduca sexta* and *D. melanogaster* [37,61]; we also used additional primers: *Ddc-ro* 5'-TCATGAGGTAGCGGTACTCGG-3' and *Ddc-fi* 5'-CAAGCTCATTCGTCTGTCGAG-3', A. Tobler pers. comm.). *Ddc-fo* and *Ddc-ro* primers amplify a region homologous to the *Drosophila melanogaster Ddc* gene between positions 1492-1928 (GenBank NM078876). In *Heliconius* this region spans two introns.

The 25µl reactions for *Co* used 2µl of crude DNA extract, 1x buffer, 2 mM $MgCl_2$, 0.8 mM dNTPs, 0.5mM of each primer and 0.025u/µl of Amplitaq polymerase. Both

pairs of primers used a cycling profile of 94°C for 1 min., then (48°C for 45 sec. and 72°C for 60 sec., 4 cycles), followed by (94°C for 45 sec., 52°C for 45 sec. and 72°C for 1min. 30 sec.) for 29 cycles. PCR products were electrophoretically separated on 1.5% low melting point agarose with ethidium bromide (1µg/ml). Bands were cut from the gel and dissolved in gelase.

Nuclear sequences were amplified in 25µl reactions containing 2µl of crude genomic DNA extract, 1x buffer, 3mM $MgCl_2$, 0.8mM dNTPs, 0.5mM of each primer, and 0.03u/µl of *Taq* gold polymerase (*Tpi*), or Amplitaq (*Mpi*, *Ci*, and *Ddc*). *Tpi* was amplified using the following step-cycle profile: 94°C for 7 min., then (94°C for 45 sec., 58°C for 45 sec., 72°C for 1min. 45 sec.) for 10 cycles with the annealing temperature reduced 0.5 °C per cycle, then 25 cycles with an annealing temperature of 53 °C. *Mpi* amplification used a profile of 94°C for 3 min., then (94°C for 40 sec., 55°C for 40 sec, 72°C for 45 sec.) for 34 cycles. *Ci* amplification used a profile of 94°C for 2 min., then 35 cycles of (94°C for 30 sec., 58°C for 1 min and 72°C for 45 sec.).

***Dopa decarboxylase* locus**

We amplified and sequenced a fifth locus, *Dopa decarboxylase* (*Ddc*); amplification used a profile of 94°C for 2 min., then 30 cycles of (94°C for 30 sec., 53°C for 1 min. and 72°C for 45 sec.). *Ddc* proved hard to amplify, and serial PCR amplifications were sometimes necessary to obtain sufficient product for cloning. Such a technique is liable to fix multiple PCR errors and is hardly recommended when accurate sequence information is required. *Ddc* sequences obtained were deposited in GenBank under accession nos. AY437778-AY437804. It was particularly difficult to amplify *Ddc* from *H. cydno*. The two partial sequences eventually obtained from *H.*

cydno proved particularly anomalous. Sequences amplified successfully *in toto* from *H. melpomene* (AY437780-AY437802) were reasonably close to those amplified from *H. himera* (AY437778, AY437779), while shorter sequences amplified with internal primers from *H. cydno* (AY437803, AY437804) and those from *H. numata* (Mathieu Joron, pers. comm.) were similar to each another, but strongly divergent from those of *H. himera* and *H. melpomene* (Additional File 1, Fig. 1). *Heliconius melpomene* is well known on morphological, bionomic and genetic grounds to be much more closely related to *H. cydno* and *H. numata* than to *H. himera*, so genealogies based on this locus must be deemed unreliable (Additional File 1, Fig. 1), possibly because sequences amplified from *H. cydno* and *H. numata* are from a more distant gene duplicate. We therefore exclude *Ddc* from the analyses of gene flow presented in the main part of this manuscript.

Additional file 1 – Table 1. Uncorrected average pairwise divergence per base pair (on and above diagonal), and net pairwise divergence (below diagonal) for *Ddc*. Numbers in brackets are the results of analysis aligning short *H. cydno* sequences and their counterparts in *H. melpomene* only.

Ddc		*H. melpomene*		*H. cydno*
		Panama	French Guiana	Panama
H. melpomene	Panama	0.0126 (0.0193)	0.0078 (0.0161)	- (0.0744)
	French Guiana	0.0059 (0.0000)	0.0057 (0.0113)	- (0.0638)
H. cydno	Panama	- (0.0608)	- (0.0631)	- (0.0062)

Additional file 1 Figure 1 – Inferred genealogy for *Ddc* locus

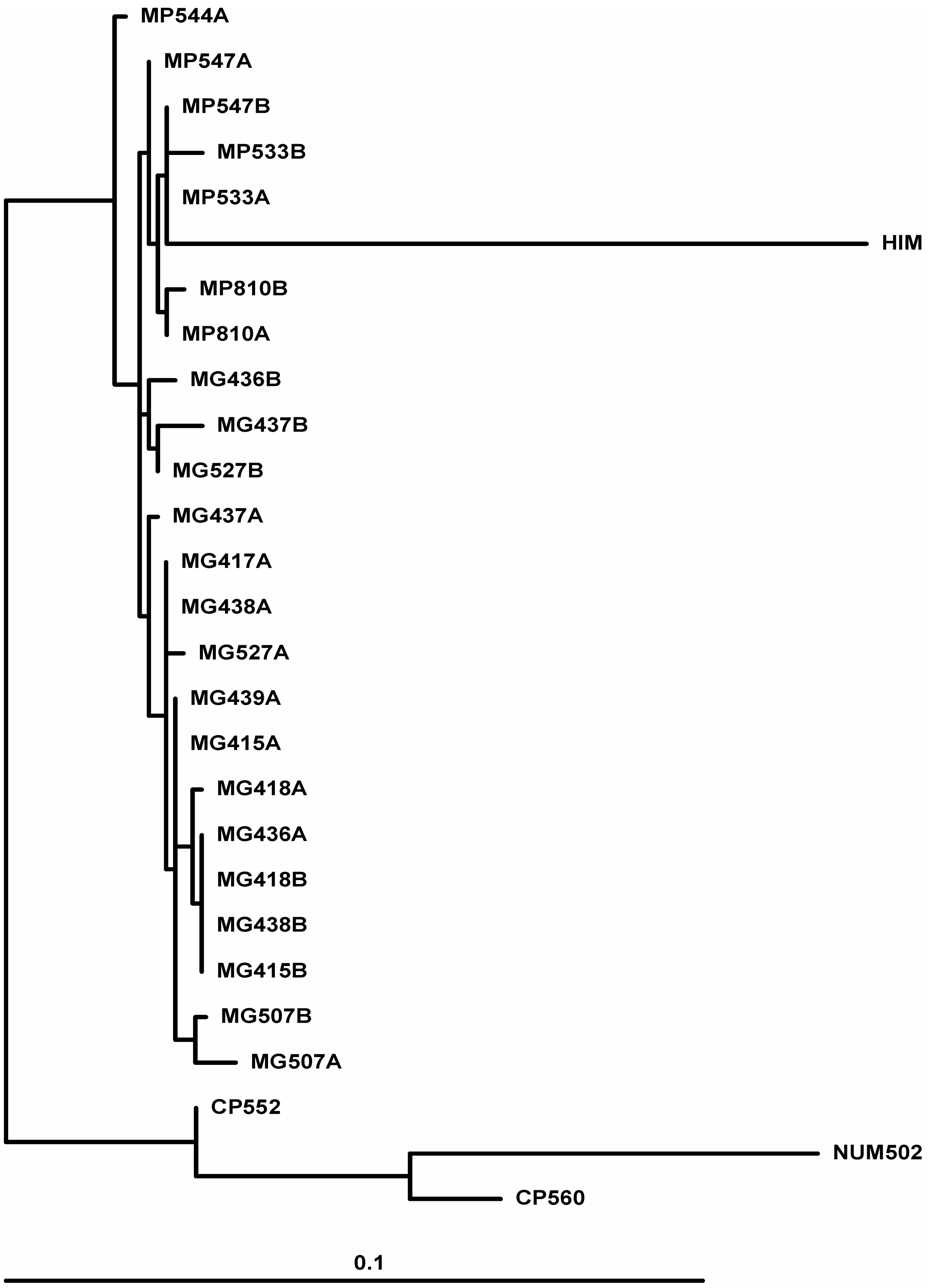

Legend as for Figs 1-4. This genealogy shows strong grouping of *H. melpomene* sequences with *H. himera* (HIM), while *H. cydno* sequences group with *H. numata*. It is clear from morphological and other genetic data that *H. melpomene*, *H. cydno* and *H. numata* are more closely related to one another than any of these is to *H. himera*. Therefore, we suspect that a different locus has been amplified in *H. cydno* and *H. numata*, and we have excluded this data from the introgression analysis.
